# Examining Canadian Equine Industry Participants’ Perceptions of Horses and Their Welfare

**DOI:** 10.3390/ani8110201

**Published:** 2018-11-07

**Authors:** Cordelie DuBois, Lindsay Nakonechny, Emilie Derisoud, Katrina Merkies

**Affiliations:** 1 Department of Animal Biosciences, University of Guelph, Guelph, ON N1G 2W1, Canada; cdubois@uoguelph.ca (C.D.); ljnakone@gmail.com (L.N.); 2 Campbell Centre for the Study of Animal Welfare, University of Guelph, Guelph, ON N1G 2W1, Canada; 3 Agrocampus Ouest, 65 rue de Saint-Brieuc, CS 84215, 35042 Rennes CEDEX, France; emilie.derisoud@gmail.com

**Keywords:** equine welfare, welfare perception, Canadian equine industry, online survey, affective states, human behaviour change

## Abstract

**Simple Summary:**

The Canadian equine industry is a diverse and fragmented industry containing a multitude of individuals whose different backgrounds and involvements shape their perceptions of the horses they use and work with. An online survey distributed to adult members of the Canadian equine industry (n = 901) was used to provide insight into participants’ perceptions of horse sentience and the welfare status of animals in the industry. Participants strongly believed that horses can experience emotions such as pain and fear, but these opinions were rarely reflected in their answers regarding welfare issues. Participants involved in disciplines having a history of using horses for work were more inclined to consider horses livestock, and this belief impacted their responses to welfare concerns, for example being less concerned about horses at auctions. While there was strong agreement regarding the welfare threats to horses in the industry, participants were more divided regarding the optimal ways to address these issues and which horses were most affected by them. Understanding these perceptions may be useful in the future to help direct educational programs and industry-wide initiatives, particularly in the area of equine welfare, in an effort to better the lives of horses through targeted knowledge transfer.

**Abstract:**

The diversity of the Canadian equine industry makes determining baseline attitudes and beliefs a challenge. Adult members of the Canadian equine industry (n = 901) participated in an online survey to report demographic information and views on the role of horses and their ability to experience affective states. Questions regarding the welfare state of all horses in the industry, potential ways to address welfare issues, and eight short scenarios were presented. Qualitative analysis, descriptive statistics, and a Chi-squared test for independence examined survey results and potential relationships. Participants strongly believed horses were capable of feeling positive and negative emotions, particularly pain and fear, but rarely were these beliefs reflected in their answers regarding aspects of equine welfare, which may be due to the large bias in these beliefs. Lack of knowledge and financial difficulties were noted as the biggest threats to equine welfare. Overall, there was widespread agreement regarding the presence of welfare issues within the equine industry, but opinions were more divided regarding how to best address them and which horses were most at risk. Understanding these perceptions may be useful to direct educational programs and industry-wide initiatives to address equine welfare through human behaviour change.

## 1. Introduction

The diversity of the Canadian equine industry was keenly underlined in the Industry Profile Study [1], not only with respect to the people within the industry, but also their involvement and the multiple ways in which they interact with and utilize horses. Just as horses serve many purposes, industry participants play numerous roles in the care and management of equids, ranging from day-to-day upkeep to operating businesses which sell feed and equipment [1]. Moreover, participants may be involved in different disciplines (e.g., English, Western) and sectors (e.g., the horsemeat industry), further adding to the diversity and fragmentation among industry groups. Awareness of the role this diversity plays in industry participants’ perceptions and beliefs is vital to understanding the human element when examining what constitutes “good” equine welfare. This type of information cannot be gleaned from animal-focused welfare assessments and so, as stated by Lund et al. [2], “Where human and animal interactions occur … the social sciences should be part of the collaborative effort” (p. 47). 

Numerous studies have examined the opinions and perceptions of horse industry enthusiasts and professionals in many different countries all related to the discussion of equine welfare. Qualitative research methods in the form of surveys and interviews have been used to document opinions regarding valuable horsemanship skills [3], the prospect of an on-farm welfare assessment [4,5], the perception and management of stereotypic behaviour [6], and to identify welfare issues and potential solutions in multiple countries (e.g., Great Britain [4]; Netherlands [7]; Italy and Germany [8]; USA [9]). Additionally, researchers have sought to determine the role that demographic factors play in the perception of issues by equine industry participants, including discipline [10], gender and agricultural background [11]. All of these studies highlight not only the differences in opinion held by participants across the different sectors of the equine industry, but also the differences in the value they place on equine welfare issues. The beliefs held by Canadian equine industry participants with regards to welfare have only recently been examined, clearly showing a lack of consensus on welfare issues and how to address them [12], and in light of the differences among equine industries, these warrant attention. 

Though the potential for horses to experience compromised welfare at the hands of human caregivers has been well-documented [13], the link between attitude and action is not yet clearly defined. For example, work done in the area of consumer perspectives and willingness to pay highlight this type of disconnect. Typically, the costs of “welfare-friendly systems” are greater than those of more traditional systems, which increases the cost of the animal product [14]. While consumer surveys indicate that a high value is placed on animal welfare, this belief may not be supported by differences in product sales [15]. In essence, while consumers value animal welfare, they are not always willing or able to pay more for products raised in welfare-friendly systems. Similarly, differences in views and behaviours have been frequently examined using short scenarios or “vignettes” to differentiate between what medical professionals would do “in theory” versus “in practice” (see review by [16]). This type of method has been used to better understand how industry participants and professionals view animal welfare and to examine perceptions in the Irish [17] and Canadian equine industries [18]. 

With respect specifically to horse owner behaviour, Hemsworth et al. [19] commented on the lack of research supporting a link between owners’ attitudes and the welfare of their animals, though they suggested that the role a horse played (e.g., companion, livestock) would affect how it was managed. Determining the extent to which horse owner beliefs are reflective of behaviour is, therefore, very important if education is meant to be the primary method of improving equine welfare. If owners who are more educated regarding welfare-friendly practices still support and utilize practices which are not welfare-friendly, then providing more education and resources may not result in meaningful behaviour change. Moreover, determining existing attitudes regarding horses can highlight areas where knowledge dissemination is poor within the industry, as well as set directions for future research and comparative analyses between perception of welfare and the welfare of animals as reported by objective assessments. The importance of this is particularly significant for horse farm owners, as participants who own horses do not necessarily own the farm in which they are kept (80% reported horse owners vs. 27% reported farm owners [1]).

Qualitative research is a useful approach to gain an understanding of perceptions, opinions and motivations of participants which can then help to guide appropriate interventions for behaviour change. As part of a larger survey project examining attitudes of Canadian equine enthusiasts, participants were asked a variety of multiple choice and open-ended questions regarding their opinions on welfare issues within the industry. Demographic information, as well as perception of the horse’s capability to experience affective states, was collected and used to determine if a relationship existed between what participants believed horses can feel and their answers to subsequent welfare-related questions. In this way, the objective of this project was not only to report on the opinions of Canadian equine enthusiasts but further examine the relationship between the perception of welfare issues in theory and in practice, using situational examples. 

## 2. Materials and Methods 

### 2.1. Survey Creation and Distribution

This study was approved by the Research Ethics Board (REB) of the University of Guelph (REB #15JA040) for the ethical use of humans in research. Potential participants (Canadian citizens with an interest in horses who were at least 18 years of age) were recruited through national and provincial equine organizations (e.g., Equine Canada), online forums, poster advertisements, and the University of Guelph REB website. A consent form was provided prior to the survey which informed participants of their right to withdraw consent at any time. The survey was hosted online using the Survey Gizmo platform and was open from 15 March 2015 to 1 May 2015.

Scientific literature and the National Farm Animal Care Council’s (NFACC) Code of Practice for the Care and Handling of Equines [20] were used as the basis for the survey’s content. All questions were reviewed by academic and non-academic peers, some of whom were not involved in the equine industry. The first 20 questions were designed to collect demographic information from the participants including: gender, age, provincial location, education, equine education, household income, type and length of involvement within the horse industry, and involvement in discipline(s). The remaining 19 questions of the survey focused on horse sentience (e.g., Do you agree or disagree that horses can feel love), welfare issues (e.g., What is the best way to assess horse welfare), and sample scenarios (described in Table 1). Where relevant, the survey filtered responses based on whether or not the participant was a horse owner. Questions were presented in the form of Likert scales, multiple-choice options, or allowed open-ended responses (see full survey in Supplementary Materials S1).

### 2.2. Survey Section: Horse Sentience

Participants were presented with five statements regarding the role of horses (e.g., as livestock, as companions, as friends) and asked to evaluate them using a Likert-scale (1 = “strongly agree” to 5 = “strongly disagree’). In addition, participants were presented with statements regarding a horse’s ability to experience both negative (e.g., pain) and positive (e.g., love) emotions or affective states and expressed their agreement or disagreement via a similar Likert-scale. 

### 2.3. Survey Section: Welfare Issues

Welfare was defined as “the overall physical and psychological well-being of an animal, where ‘good welfare’ is a state where an animal is free from hunger or thirst; distress and discomfort; pain, injury and disease; unnecessary suffering; and able to express normal behaviour” [21]. Participants were asked a series of questions regarding the best method of horse welfare assessment (e.g., vitals, good performance), the welfare status of horse in the Canadian horse industry (“do you believe there are welfare issues in the industry”), and the demographic horse population perceived to be most at risk (e.g., horses on acreages, horses at riding facilities). Potential answers were provided to them or they had the option to enter open text. They were also presented with a predetermined list of potential horse welfare issues (e.g., unwanted horses), causes of welfare issues (e.g., lack of financial resources), and solutions (e.g., education programs) and asked to evaluate their impact, likelihood, and effectiveness respectfully. Finally, participants were asked if certifying horse facilities as “welfare-friendly” was an effective strategy for improving welfare and, if yes, who should certify facilities (e.g., horse organizations) and how much extra they would be willing to spend on boarding costs at a welfare-certified facility. 

### 2.4. Survey Section: Sample Scenarios 

To examine perceptions of equine needs, participants were asked to evaluate a series of scenarios (Table 1) which described a situation in which a horse’s welfare might be compromised. Participants were provided with a sliding scale (with the furthermost left side indicated as 0 = “poor welfare” and the furthermost right as 100 = “good welfare”) and were instructed to slide the cursor to the point which best described their perception of the welfare of the horse. 

### 2.5. Statistical Analysis 

A post-hoc power analysis was performed to determine the impact of the sample size using a small observed effect size (Cohen’s d = 0.2) and a probability level of 0.05. Answers provided in any multiple-choice question with an “other” option or in the open-ended questions were structurally coded using QSR International’s NVivo 11 qualitative data analysis Software (Version 11.3.1 777, QSR International Pty Ltd., Burlington, MA, USA). Coding categories were drawn directly from single words or phrases used by participants. In the case of questions which asked participants to describe their involvement in the industry (equine education, industry involvement, and discipline involvement), pre-existing categories were collapsed for a more streamlined analysis and some additional categories were created based on open-ended responses from participants. This was due to the large number of available categories initially provided to respondents. 

Descriptive analysis was conducted using IBM Corp’s SPSS Statistics for Windows Version 24.0 (IBM Corp., Armonk, NY, USA, 2016). A Chi-squared test for independence (IBM Corp.’s SPSS Statistics for Windows Version 24.0) was used to examine potential relationships between demographic factors and opinions regarding horse sentience and related questions from welfare-focused sections. Some categories were collapsed in the event of low counts (<5). Data provided in the “other” category of multiple-choice questions was reported on but not included in chi-squared tests due to low counts. In the case of significant (*p* > 0.05) 2 × 2 Chi-squared tests, Fisher’s Exact test was used to determine directionality. A Spearman’s Rank correlation was used to compare grouped demographics (e.g., age group) to a participant’s scenario score for each of the eight scenarios. For binary demographic variables (e.g., knowledge of the Code of Practice), a one-way ANOVA was to determine if these statistically affected a participant’s scenario score. 

## 3. Results

### 3.1. Demographics

**Figure 1:**
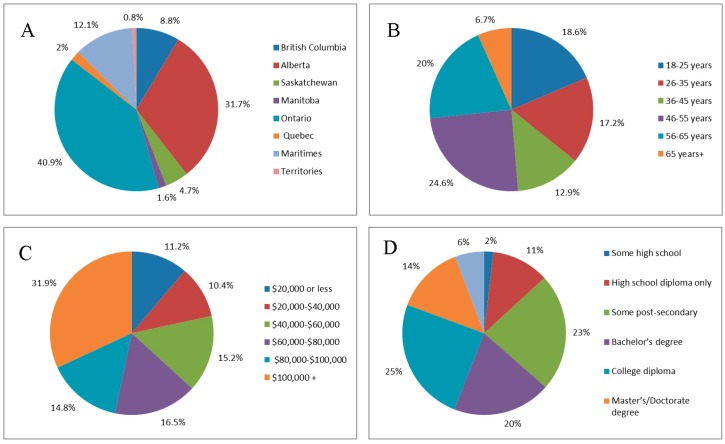
Pie graphs illustrating the spread of participants (n = 901) in the demographic categories of (**A**) Canadian province, (**B**) age, (**C**) annual income, and (**D**) education level.

The estimated population of horse enthusiasts in Canada is 550,000 [1], thus the 901 survey participants represented 0.16% of the population, giving a post-hoc power level of 0.82 for the analyses. The demographic spread of participants (n = 901) with respect to age, home province, income, and education can be found in Figure 1. The typical respondent for this survey was female (92.5%, n = 831/898), located in Ontario (40.8%, n = 366/894) or Alberta (31.7%, n = 283/894), over the age of 45 (44.6%, n = 462/901), had an annual income of at least $60,000 (63.2%, n = 529/837) and had some form of post-secondary education (81.1%, n = 728/898). With respect to formalized equine education, 22% (n = 198/901) of participants had completed college or university courses, with fewer participants having completed rider certification through Equine Canada (18.2%, n = 110/901) or equine diplomas (12.2%, n = 164/901). 

Involvement in the industry was diverse, with individuals participating in at least one of the twenty-one options provided (see survey Q7, App S1). The greatest participant involvement was by those who volunteered for equine organizations or considered themselves equine activists (30.7%, n = 277/901), followed closely by individuals who were involved in non-racing competitions (29.5%, n = 266/901) and as general riders (25.1%, n = 266/901). The racing industry and those individuals involved in regulatory aspects of the industry (e.g., animal welfare enforcement) made up the smallest proportion of participants, at 3.2% (n = 29/901) and 1.2% (n = 11/901) respectively. Most participants (78.2%, n = 683/874) had been in the industry for more than ten years. Discipline involvement showed a similar diversity, with participants involved in at least one of the twenty-five options provided (see survey Q9, App S1). Participants were predominantly from English (61.8%, n = 557/901; included: English pleasure, eventing, dressage, show jumping, hunter, fox hunting, and endurance) and Western disciplines (40.5%, n = 365/901, included: Western pleasure, cutting, barrel racing, cattle penning, reining, roping, rodeo, mounted shooting, trick riding, outfitting, and trail riding).

Of the five options regarding horse care knowledge, participants were most familiar with body condition scoring (BCS; 78.6%, n = 708/901) and the Canadian Code of Practice for the Care and Handling of Equines [20] (53.9%, n = 486/901). Participants were less familiar with the American Association of Equine Practitioners Lameness Scale [22] (35.6%, n = 321/901), the Five Freedoms [21] (29.7%, n = 268/901), and Equitation Science [23] (20.4%, n = 184/901).

### 3.2. Horse Sentience

**Figure 2:**
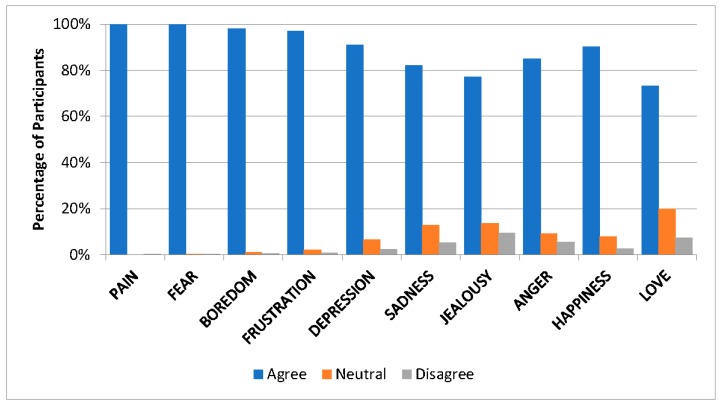
Viewpoints of participants (n = 901) measured using a Likert-scale (agree, neutral, disagree) regarding the ability of horses to feel various affective states. Responses of “strongly agree” and “agree” were combined, as were “strongly disagree” and “disagree”.

Participants most commonly agreed or strongly agreed with the statements that horses were companion animals (89%, n = 679/759) and friends (67%, n = 507/752). Nonetheless, 53% (n = 402/753) of respondents still considered horses livestock. Participants involved in Western disciplines were more likely to believe that horses were livestock (Fisher’s Exact Test [FET] two-sided *p* = 0.015), but less likely to consider horses companions (FET two-sided *p* = 0.033) or friends (FET two-sided *p* = 0.028). Similar results were seen for individuals involved in breed-related (e.g., breed shows; FET two-sided *p* < 0.01), driving (FET two-sided *p* = 0.019), and in-hand (e.g., halter; FET two-sided *p* < 0.01) disciplines, who were all more likely to consider horses livestock. In contrast, participants involved in the English discipline were more likely to consider horses companions (FET two-sided *p* < 0.01) and friends (FET two-sided *p* = 0.025). The majority of participants agreed or strongly agreed that horses could feel a wide variety of affective states, with very few respondents believing that horses could not experience frustration, depression, sadness, jealousy, anger happiness and love (Figure 2). 

### 3.3. Welfare Issues

Participants almost unanimously (97%, n = 659/679) agreed that there were welfare issues within the Canadian industry, but opinions were divided as to which groups of horses were at risk for welfare issues. Horses at auctions or feedlots received the highest consensus agreement (65%, n = 582/901), with agreement ranging from 9% (all horses, n = 82/901) to 50% (competition horses, n = 448/901). Horses with “ignorant owners” and horses intended for slaughter were also suggested as specific groups of horses at risk. When examining specific welfare issues, the majority (>70%, n = 449/642) of participants agreed or strongly agreed that unwanted horses, horses that were not trained appropriately, inappropriate or lack of horse knowledge by owners or caregivers, the financial costs of horse care, ineffective or lack of horse welfare laws, and ineffective or lack of enforcement capacity to protect horse welfare all contributed to poor horse welfare. Participants had more divided responses regarding horse slaughter, with 68% (n = 437/646) believing horse slaughter was a welfare issue while 56% (n = 350/630) believed not having slaughter as an option was a welfare issue. 

Chi-squared analyses indicated an association between participants’ views on horses and their answers with regards to what horses were affected by welfare issues and what issues could be considered welfare issues. Participants who considered horses to be livestock were more likely to be concerned about rescue horses (FET two-sided *p* < 0.01) and all horses (FET two-sided *p* = 0.017), but less likely to be concerned about horses at auction (FET two-sided *p* = 0.004). In contrast, participants who consider horses companion animals were more likely to be concerned about breeding stock (FET two-sided *p* < 0.01), competition horses (FET two-sided *p* = 0.043), farm horses (FET two-sided *p* = 0.015), and horses at auction (FET two-sided *p* < 0.01). Finally, respondents who believed that horses were friends were more likely to be concerned about horses at auction (FET two-sided *p* = 0.015). 

When asked to rank the potential causes of reduced horse welfare in terms of frequency, 41% (n = 233/569) of participants ranked ignorance as being the most frequent and 55% (n = 310/565) of participants ranked malice as being the least frequent. Lack of financial resources and traditional practices most commonly ranked in the top four (77% (n = 439/571) and 62% (n = 350/569) respectively), while pride and lack of time by horse owners or staff most commonly ranked in the bottom three (53% (n = 300/567) and 63% (n = 356/569) respectively). Greed was the most divided, with 36% (n = 204/569) of participants ranking it in the bottom three while 49% (n = 277/569) of participants ranked it among their top three causes. Education programs about standards of horse care were most frequently ranked first in effectiveness for addressing welfare issues (67%, n = 265/632), while low stress and humane handling training for horse professionals most frequently ended up in the lowest rank (42%, n = 196/461). Opinions were varied for the other potential strategies for improving welfare. 

A participant’s possession of university-provided horse education statistically impacted their ranking of education programs as a potential solution to welfare problems (χ^2^(5) = 17.302; *p* = 0.004, n = 901). Consensus was difficult to achieve regarding who should deliver educational programs, with the majority of participants indicating horse organizations (67%, n = 605/901) and veterinarians (59%, n = 533/901) being the best groups to be in charge of these programs. Participants also suggested academic researchers, 4H clubs, and experienced horsemen could fulfill this role. Participants with university-provided horse education were more likely to choose government organizations (χ^2^(1) = 6.831; *p* = 0.009, n = 901), horse organizations (χ^2^(1) = 7.262; *p* = 0.007, n = 901), researchers (χ^2^(1) = 6.027; *p* = 0.014, n = 901), and veterinarians (χ^2^(1) = 5.467; *p* = 0.019, n = 901) to deliver education programs (FET two-sided *p* < 0.01). 

A similar divide was seen when participants were asked who should grant welfare certifications to facilities. Though 70% (n = 460/658) of participants indicated that they believed a certification would improve equine welfare, only 57% (n = 255/450) believed the program should be delivered by a horse organization. A participant’s opinion on who should grant welfare certification was only statistically affected by their involvement in the industry as a rider (χ^2^(1) = 3.837; *p* = 0.050, n = 450), with riders more likely to choose a horse organization (FET two-sided *p* = 0.031). The majority of participants (66%, n = 359/450) indicated they would pay between $1–100 extra per month to a facility that was welfare certified. Annual income had a significant association with the amount participants were willing to pay to board their horses at a welfare-certified facility (χ^2^(15) = 26.878; *p* = 0.030, n = 429) but there was no clear trend (i.e., those with a higher income were not always the ones willing to pay more).

### 3.4. Sample Scenarios 

**Figure 3:**
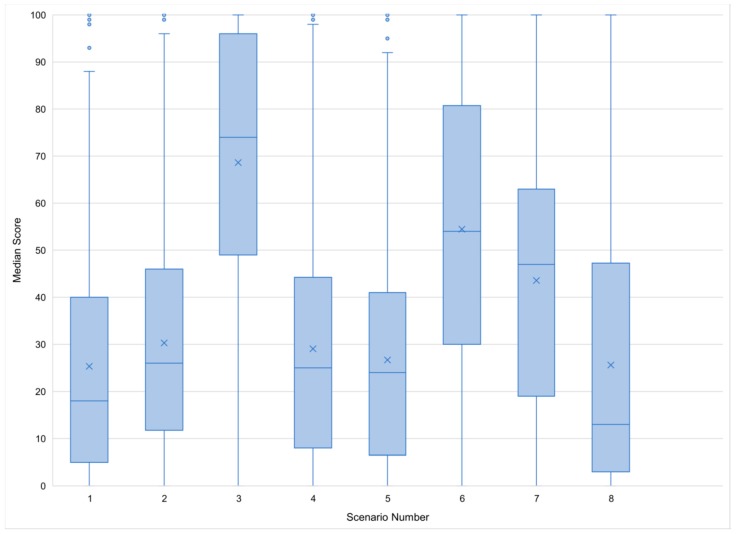
Box and whisker plot of scenario (eight numbered scenarios) scores as assigned by survey respondents (n = 901). Vignettes were scored between 0 (poor welfare) and 100 (good welfare) on a sliding scale.

Participants provided scores for each of the eight scenarios, with a score of 0 indicating poor welfare and a score of 100 indicating excellent welfare. Participants considered scenario 1 (horses kept in a pasture during winter months) and 8 (horse sedated prior to training) to be those which had a horse experiencing the worst welfare, with median scores of 18 (Inter-Quartile Range [IQR] = 35) and 13 (IQR = 44) respectively (Figure 3). Conversely, participants believed that horses in scenarios 3 (an injured horse under veterinary supervision) and 6 (whip use during training) had the best welfare with median scores of 74 (IRQ = 47) and 54 (IQR = 50.25) respectively. 

Though some significant correlations occurred between demographic variables and a participant’s scenario score, all correlations were very weak (*r* < 0.19). Female participants scored scenario 1 lower on average (F(1,616) = 7.004, *p* = 0.008), and horse owners scored scenario 6 higher on average (F(1,632) = 4.236, *p* = 0.040). Participants who were aware of the Five Freedoms scored scenarios 1, 4, 5, 6, and 8 higher on average, and scenarios 3 and 7 lower on average (e.g., Scenario 4; F(1,612) = 14.096, *p* < 0.01). Individuals who worked directly with horses scored scenarios 6 (F(1,631) = 5.302, *p* = 0.022) and 7 (F(1,615) = 8.491, *p* = 0.004) higher on average. Knowledge of the Code of Practice only impacted scenario 4 (F(1,612) = 10.938, *p* = 0.001), with participants who were familiar with the Code of Practice scoring the scenario higher on average. 

## 4. Discussion

### 4.1. Demographics

Demographically, the survey population is comparable to that of the 2010 Equine Canada Industry Profile Study [1], with the average participant being female, over the age of 45, having an annual income of at least $60,000 having some form of post-secondary education, and being mainly horse owners and/or horse riders. Additionally, the provinces of Ontario and Alberta are represented by the highest number of participants and also support the largest percentage of the Canadian equine industry [1]. The largest demographic bias in the data was represented by the dominance of female participants. This, however, is not surprising. Much greater numbers of female than male participants in surveys regarding equine welfare have been reported in the United States of America [24], Canada [25], the Netherlands [7], as well as general animal welfare surveys [5,26]. As a result, as well as the fact that there is a high proportion of female participants in the Canadian equine industry [1], this bias was determined to not significantly affect the outcome of the survey. The diversity of discipline and industry involvement are also comparable to the 2010 Equine Canada Industry Profile Study [1]. Overall, this subset of the Canadian equine industry is, demographically speaking, considered to reflect the nature of the industry. 

There are, however, limitations to this method of surveying participants. The study sample represented approximately 0.16% of the Canadian adult horse enthusiast population (approximately 550,000 adults, [1]), a relatively small proportion of individuals. Furthermore, participation was voluntary, and those individuals who had an interest in welfare and the time required to complete the survey would have participated. As a result, certain opinions may have been over- or underestimated due to the nature of the surveying techniques. The merits of using findings from voluntary surveys have been previously discussed (e.g., [26,27,28]) and are a recognized limitation of this study. Online surveys in particular may suffer from self-selection [29] (i.e., only available to those who can access and interpret them), however the typical Canadian horse enthusiast is known to access the internet regularly and have a high level of education [1]. However, several industry groups identified in the 2010 Equine Canada Industry Profile Study are not represented in this study (e.g., horse traders) and as a result their opinions have not been recorded here. Though they make up a relatively small part of the national industry, they still play a role in the care and management of horses. 

With respect to the participants’ knowledge, the majority of participants believed that they were at least somewhat knowledgeable with respect to horse care. Despite this, the majority of participants were unfamiliar with most of the document options presented to them. Perhaps the most concerning is that fewer than 55% of participants knew about the NFACC Code of Practice, a national document outlining the equine industry standards. DuBois et al. [30] also found a similar result when surveying managers involved in an on-farm welfare assessment, which questions the reach of this important code. This, and the disparity between perceived horse knowledge and the reporting of horse knowledge using more scientific-based terminology, may reflect the difficulty in information dissemination. The poor perception of scientific information has been reported to be a barrier to not only the movement of information but also the uptake of new practices [31], and this may be a strong contributing factor to the lack of awareness of published research or compiled documents. 

### 4.2. Horse Sentience

Horses were predominantly considered companion animals with the vast majority of participants strongly believing that horses could feel all affective states (particularly pain, fear, and boredom). While the physical effects of pain and fear may be objectively assessed [32], the capability to feel love requires a belief in the higher capacities of emotion. This belief that horses can feel love is not unique to Canadian horse enthusiasts as others have reported an open willingness to relate stories of companionship and friendship with horses [33,34], and recent research shows similar results from Brazilian horse owners and caretakers [35]. Humans in general easily attribute emotions to all animals, and horse owners in particular attributed jealously to horses more often than non-horse owners [36]. It is worth noting that discipline played a significant role in an individual’s perception of a horse’s role. Participants who were involved in disciplines which have a history of using horses for work (Western, driving) or who show horses in a manner similar to other livestock (breed shows) were more inclined to consider horses livestock. In contrast, participants whose discipline is focused on the use of the horse predominantly for riding as a hobby or sport were more likely to consider them companions. The intersection between discipline and the perception of a horse’s role has not been well-studied; however, a similar situation may be visible here as in work by Rice et al. [11], who noted that welfare perceptions of horses differed between those who had grown up in an agricultural background versus those who had not. This crossroad of opinions is unique to the equine industry, and warrants further investigation into the effects of discipline and specifically information transfer through discipline leaders (e.g., horse organizations) on the perception of horses and their welfare.

### 4.3. Welfare Issues

Participants were in strong agreement regarding the presence of welfare issues in the industry and whether individual situations were welfare concerns, but they had more difficulty reaching a consensus on which groups of horses in the industry were at risk for the identified issues. While the surveys conducted by Horseman et al. [4] and DuBois et al. [12] allowed participants to suggest the groups of horses which could experience compromised welfare, the diversity of answers and differences in the number of times a particular answer was raised match the diversity of answers given by respondents of this study. Even though survey respondents may be focused on one group in particular (racing in the case of [4]) and horses in feedlots in the case of participants in this study), the belief that welfare affects horses in other disciplines is still held by many others. Lofgren et al. [10] noted in their survey of horse enthusiasts that individuals were more concerned about the welfare of horses in disciplines they were not engaged with, and a similar phenomenon may be visible here given the diversity of industry involvement and discipline. As mentioned previously, there was a lack of involvement by those individuals who work at horse auctions or feedlots, which, combined with the fact that horses were most commonly seen as companion animals or family, likely resulted in increased attention to this group of horses. Furthermore, with representatives from twenty-five disciplines, it is likely that experiences varied sufficiently to warrant differences in perception of horses raised in different conditions. 

Horses, due to the different ways in which they are used by people, occupy a unique niche in which they are not entirely companion animals or livestock. While some owners treat them the same way they might treat a cat or a dog, there are distinct differences between horses and companion animals [33]. Horses do not share their living quarters with humans, and as such must be either managed by their owner in a facility separate from the house or managed by someone else. This act of raising animals in a barn away from the house is more akin to that of livestock, which likely accounts for the differences in perception regarding what constitutes a welfare issue. For instance, those who believed that horses were companions or friends were more critical of groups of horses kept in ways similar to traditional livestock (e.g., breeding farms, horses on acreages, and horses at auction), as well as competition animals. In contrast, those who considered horses to be livestock were more critical of the welfare condition of horses at rescues and of all horses, but were less critical of horses at auctions. In the future, examining the perception of a horse’s role as well as background (as in [11]) may shed additional light on this separation of opinion. A similar type of division of concern was seen in the case of the perception of slaughter. Both slaughter and the absence of available horse slaughter are recognized as welfare issues for different reasons [37]. Given the legality of horse slaughter in Canada—and the potential repercussions of banning it [37]—this is a topic that warrants further investigation. There is a paucity of data regarding the state of the Canadian horse slaughter industry as well as the opinions of industry participants regarding slaughter and feedlot practices, which needs to be addressed before any meaningful analyses can be conducted. 

Repeated themes of lack of knowledge and the value of education programs led from within the equine industry are similar to findings from DuBois et al. [12], which suggest a strong desire to improve the welfare of the industry without government intervention. Lack of knowledge as a perceived threat to equine welfare is well-documented by researchers in other equine industries in Ireland [17], Great Britain [4], and Australia [19]. The rankings of the motivators of poor welfare by survey participants is similar to results found by both DuBois et al. [12] and Horseman et al. [4] with lack of knowledge and financial difficulties emerging near the top of the list. Results regarding what methods would best address welfare issues were also similar to responses given by Canadian equine professionals [12], particularly with respect to preferring education from inside the industry over government intervention. Whether or not participants had academic equine education affected their belief in the usefulness of education as a method of improving welfare. Given the strong belief that lack of knowledge plays an important role in causing welfare issues, the natural inclination would be to choose education as the method to improve this. 

Providing education may not be enough to change the way people manage horses, as studies have shown that behaviour change to incorporate new knowledge into practice did not occur [38]. With new research findings, implementation of best practice relies not only on educating people, but on human behaviour change. Behaviour change is difficult to embrace, but not impossible, as seen in numerous examples of improved health when significant lifestyle changes were implemented [39]. However as simple a behaviour change such as taking a daily medication to prevent reoccurrence of a heart attack showed very low rates of adherence over the long term [39]. In spite of having the knowledge of what is best, people still resort to practicing what they know [40]. It is essential to recognize what the drivers of change are for a specific population before successful interventions can be implemented to change behaviour patterns. Interventions can take various approaches (physical, psychological, social, etc.) but require capability, motivation and opportunity to be enacted [40]. Education alone may not provide the best answer, but could be combined with other approaches such as incentivisation, persuasion, training, restriction, coercion, or modeling [40]. Participants in this survey more frequently attributed welfare issues to ignorance rather than malice, suggesting that they believe people lack the knowledge, not the desire, to improve welfare. Behaviour change in management methods to improve welfare of horses may be possible as the capacity, opportunity and motivation to learn are present. 

### 4.4. Sample Scenarios

Despite the wide spread of scenario scores, five out of eight of the scenarios received a median score below 40/100, which suggests participants believed welfare to be compromised in the majority of the scenarios. The scenarios which received the lowest median scores, thus considered the most welfare-compromising, involved horses being pastured overwinter without water and the scenario in which a horse is given a sedative prior to training. Snow is recognized as inadequate source of water for horses in the NFACC Code of Practice [20], and this belief appears to be widely held by participants of this survey, with female participants ranking this scenario more of a welfare concern on average than male ones. With respect to the sedative drug, horse enthusiasts in this survey were more critical of this scenario than equine professionals were of a similar scenario [12]. While professionals in DuBois et al.’s study were asked to rank scenarios on a 0 to 5 scale (versus the 0 to 100 sliding scale provided in this study), the scenario in which a horse was given a sedative prior to a competition only a received a median score of 3 [12] and it was not the scenario with the lowest median score. Opinions on drug use (or abuse) in horses is understudied, but sedatives appear to have a negative connotation in this group of equine enthusiasts. An open-ended section where participants could justify their answers (as in [12]) may have illuminated why participants felt so strongly about this situation. 

The scenario which received the highest median score (scenario 3; an injured horse under veterinary supervision) was scored considerably higher (indicating better welfare) than all other scenarios. While it is possible that stall rest may compromise a horse’s welfare due to potential social isolation and limited access to free movement, the horse is still being provided with some exercise and is being rested (presumably) so that its injury can heal. This may have accounted for the score being so high as horse enthusiasts have been shown to accept a temporary compromise in welfare in exchange for better welfare in the future [12]. Horseman et al. [4] also noted that participants in their study downplayed any welfare compromises they had to make in their own management styles, which may also factor into this scenario’s score. Finally, veterinarians are widely considered to be an important source of information about care by horse owners [41] and as such the designation of the horse as “under veterinary supervision” may have encouraged participants to award it a higher score. 

Though scenario 6 (whip use during training) received the second highest median score (54), this value is near the middle of the scale (0–100) provided to participants, indicating no clear opinion on whether artificial aid use is welfare compromising or not. Previous results have shown that Canadian horse enthusiasts use whips primarily to augment (e.g., leg) cues. In that study, it appeared that riders were correctly using the whip for negative reinforcement to amplify the leg aid [25]. The use of artificial aids, particularly whips, as a means of discipline rather than a reinforcement of forward locomotion can cause problems if used incorrectly [42]. Additionally, the use of both whip taps and a neck pats may result in unclear signaling, which can compromise equine learning [43]. As a result, this situation may cause frustration in the horse, which perhaps was not considered as welfare threatening to some participants as it was to others. In this instance, how much value the participant put on emotional stress versus physical stress may have contributed to the score they awarded this scenario. Horse owners and those who worked directly with horses scored this scenario higher on average, which may be a result of their familiarity with such practices. A similar result was seen in [4], who noted that horse enthusiasts were more critical of unfamiliar practices, particularly when they were asked to evaluate the welfare of horses as a result of said practices. 

Of particular interest was the association between knowledge of the Five Freedoms and scenario scores, which affected all scenarios except scenario 2 (individual stalling and turnout for horse). While one might suggest that having a knowledge of basic needs of all animals would help to determine if welfare was being compromised in the scenarios, participants who knew about the Five Freedoms were the most critical (indicating compromised welfare) of the two scenarios which featured stall rest (scenario 3) and a cribbing collar (scenario 7). In turn, they scored the scenarios involving snow for water (1), deep litter bedding (4), financial distress (5), whip use (6) and tranquilizer use (8) higher on average (indicating acceptable welfare). Of note, however, is the fact that participants were asked if they were familiar with the Five Freedoms, not whether they understood them. Though the freedoms themselves are written in very straightforward language, they are not designed to be prescriptive. It is also possible that participants knew of the Five Freedoms but did not know what they entailed, or that they believed the Five Freedoms were synonymous with a more “natural living” style of animal welfare [44], which would explain why they were critical of the scenarios which most compromised this. 

Additionally, as mentioned earlier, horse enthusiasts are less critical of welfare compromises they themselves make [4], which may be the case in scenario 2. Knowledge of the NFACC Code of Practice, in contrast, only affected the survey participants’ scores in the scenario involving a deep litter bedding. It is unclear why knowledge of the NFACC Code of Practice only affected this scenario, as the code states that bedding must be clean and dry but does not indicate that deep litter bedding is unacceptable. Further examination of participants’ understanding and interpretation of both the Five Freedoms and Code of Practice may have offered insight as to why this knowledge affected answers the way it did.

## 5. Conclusions

Participants had a diverse involvement in the Canadian equine industry, and this diversity was reflected in their answers regarding the current welfare issues within the industry. Respondents acknowledged the presence of welfare issues within the industry but were divided with respect to which horses were at risk and what were the best methods to resolve welfare issues. Though participants strongly believed that horses were capable of experiencing affective states (particularly pain and fear), these beliefs were not always reflective of situations they believed compromised equine welfare. These opinions were more likely to be affected by what role participants assigned to horses and how they were involved in the equine industry. Determining how welfare compromises are made and under what circumstances could be very valuable to understanding the motivations and current state of the welfare of horses in the Canadian industry. Additionally, this information can be used to direct education programs and help target human behaviour change for different equine industry communities and disciplines.

## Figures and Tables

**Table 1:** Description of the eight scenarios of potentially compromised horse welfare used to assess survey respondent thresholds of acceptable welfare.

Scenario	Description
1	Horses are kept in a large pasture during the winter months. They are given continuous free access to a hay bale, and a new bale is provided before the hay runs out. The horses’ primary water source is from eating snow.
2	A horse is housed individually in a box stall and turned out individually into a small outdoor pen for a few hours each day. The horse can see other horses, but not interact with them. Good quality hay is provided three times a day in the stall, but no hay or grass is available in the outdoor pen.
3	A horse sustains a leg injury that requires long-term pain medication and two months of stall rest. The horse is given painkillers under veterinary supervision and is housed individually in a box stall, with one hand-led walk for 30 min per day.
4	At a barn, horses are stabled overnight and turned out into a large pasture for most of the day. The staff adds fresh straw to the stalls each day, but no manure or urine is picked out regularly, creating deep litter bedding.
5	A horse owner is experiencing financial difficulty. The owner compensates by prolonging trimming of the horses’ hooves to every 10 weeks, deworms only in spring and fall, and feeds hay once a day.
6	A trainer uses a whip to discipline young horses for misbehavior. The horses are repetitively tapped lightly with a whip until they stop the unwanted behaviour. Once they stop, the horses are rewarded with a pat on the neck.
7	A horse is cribbing the top of its stall door. To stop the horse from cribbing, a horse owner puts a cribbing collar on the horse and takes the collar off only when the horse is ridden.
8	A horse owner is backing (saddling) a young horse for the first time. To prevent any potential bucking or rearing, the owner administers a sedative to the horse prior to working with him.

## Supplementary Materials

The following are available online at http://www.mdpi.com/2076-2615/8/11/201/s1, S1—Canadian Horse Welfare Survey. S2—raw data. Note that confidentiality was ensured to participants when they answered this survey and as such demographic data are not provided.
