# Supplementary material for: Examining Canadian Equine Industry Participants’ Perceptions of Horses and Their Welfare
Source: Animals (Basel). 2018 Nov 7;8(11):201. doi: 10.3390/ani8110201 (PMC6262281; doi:10.3390/ani8110201)
Supplement: Supplementary file 1 [file animals-08-00201-s001.zip › Canadian horse welfare survey-welfare paperV.pdf]

Canadian horse welfare survey

Consent form

ID: 10

Industry views of the prevalence and perception of horse welfare issues in Canada

CONSENT TO PARTICIPATE IN RESEARCH

You are asked to participate in a research study conducted by *Dr. Katrina Merkies and Lindsay Nakonechny*, from the *Department of Animal and Poultry Science, Ontario Agricultural College* at the University of Guelph. The results of this project will contribute to the fulfillment of a graduate student major project.

If you have any questions or concerns about the research, please feel free to contact:

Dr. Katrina Merkies: Faculty Supervisor
Associate Professor
Dept. of Animal & Poultry Science
University of Guelph
Guelph, ON, Canada. N1G 2W1
(519) 824-4120 x54707
kmerkies@uoguelph.ca

Lindsay Nakonechny: MSc student
University of Guelph
(780) 819-4914
lnakonec@uoguelph.ca

PURPOSE OF THE STUDY

This survey-based study is distributed to the Canadian horse enthusiast population in order to examine the current state of the Canadian horse industry and determine

prevalence and perceptions of horse welfare issues. The objectives are to understand industry management practices, rationale and frequency for contacting veterinarians, attitudes towards horse behaviour and welfare issues, and how information is acquired. By completing an online survey, the data will be compiled to determine industry prevalence and predominant opinions of horse welfare issues in Canada. Once major areas of concern for horse welfare are identified, feasible and effective solutions can be developed to improve horse welfare in Canada.

PROCEDURES

If you volunteer to participate in this study, we would ask you to do the following things:

To take part in this survey, you must be 18 years of age and a resident of Canada. Please take the time to read each question clearly and carefully, and answer honestly and to the best of your ability. If you feel uncomfortable answering any question, you have the option to skip a question. There are no right or wrong answers. The survey should take approximately 20-30 minutes of your time.

To thank you for your help, there is an opportunity to enter a prize draw to win a two-week online education course (*choice of Behaviour & Safety, Colic Prevention, or Biosecurity course offered in Fall 2015 or Winter 2016*) offered by Equine Guelph (valued at $75 + tax)! At the end of the survey, you will be asked to provide your email address if you choose to enter the prize draw or wish to receive the results of the research. The chance of winning is estimated at 1 in 100.

POTENTIAL BENEFITS TO PARTICIPANTS AND/OR TO SOCIETY

Evaluation of the current state of the Canadian horse industry is a first step towards developing welfare-friendly horse care standards. The data will identify areas of horse welfare concern, potential strategies to improve welfare, and effective methods of information delivery on optimal care and management to horse enthusiasts. In addition, these results will help develop a Canadian-based horse welfare assessment model, which can be translated to a welfare assessment audit by third-party groups. Attention to horse welfare issues will ensure responsible on-farm and facility care of horses.

CONFIDENTIALITY

Every effort will be made to ensure confidentiality of any identifying information that is obtained in connection with this study.

IP addresses will not be collected as part of the survey. Any identifying information for the delivery of the final results or entry into the draw will be collected separately from the survey. Data from the survey will be stored on a password-protected computer and only be accessible to the Faculty Supervisor and student researcher. Information will only be retained until the results of the research are published. Please

note that confidentiality cannot be guaranteed while data are in transit over the internet. Participants should clear the browsing history, cache and cookies and log off their computer to help ensure confidentiality, particularly when using a public computer. This can be done in Internet Explorer by selecting *Tools – Internet Options – Delete Browsing History* (on the General tab).

PARTICIPATION AND WITHDRAWAL

You can choose whether to be in this study or not. If you volunteer to be in this study, you may withdraw at any time before submitting the survey without consequences of any kind by closing your browser. All data entered up to the point of withdrawal will be permanently removed. You may also decline to answer any specific questions you are uncomfortable with and still remain in the study. Once you have completed the survey and clicked "submit", no identifying information will be connected with your submission and you will be unable to withdraw.

RIGHTS OF RESEARCH PARTICIPANTS

You may withdraw your consent at any time prior to completing the survey and discontinue participation without penalty. You are not waiving any legal claims, rights or remedies because of your participation in this research study. It is recommended that you print this consent form for your records. This study has been reviewed and received ethics clearance through the University of Guelph Research Ethics Board. If you have questions regarding your rights as a research participant, contact:

Sandra Auld
Director, Research Ethics
University of Guelph
437 University Centre
Guelph, ON N1G 2W1

Telephone: (519) 824-4120, ext. 56606
E-mail: sauld@uoguelph.ca
Fax: (519) 821-5236

SIGNATURE OF RESEARCH PARTICIPANT/LEGAL REPRESENTATIVE

By clicking "*next*" below, you accept that you have read the information provided for the study "*Industry views of the prevalence and perception of horse welfare issues in Canada*" as described herein. All questions have been answered to your satisfaction, and you agree to participate in this study.

Please print this page for your records

Demographics

The term "horse" within this survey refers to all equids including horses, ponies, donkeys, and mules.

ID: 9

1) Select your gender:

( ) Male
( ) Female
( ) Other

ID: 11

2) Select your age category:

( ) 18-25 years
( ) 26-35 years
( ) 36-45 years
( ) 46-55 years
( ) 56-65 years
( ) 65 years or more

ID: 12

3) Which province or territory do you reside in?

( ) British Columbia
( ) Alberta
( ) Saskatchewan
( ) Manitoba
( ) Ontario
( ) Quebec
( ) New Brunswick
( ) Prince Edward Island
( ) Newfoundland and Labrador
( ) Nova Scotia
( ) Yukon
( ) Nunavut
( ) Northwest Territories

ID: 13

4) What is your highest level of education?

( ) Some high school
( ) High school diploma
( ) Some university or college

( ) College diploma

( ) Bachelor's degree

( ) Masters or doctorate degree

( ) Professional certification (eg. Board-certified veterinarian)

ID: 14

5) Do you have any formal equine education (check all that apply)?

[ ] College or university courses

[ ] Equine diploma or certificate

[ ] Equine veterinary medicine

[ ] Coaching certification

[ ] Rider level program

[ ] 4H or Pony Club

[ ] Other, please list:: ____

ID: 15

6) What is your annual household income?

( ) $20k or less

( ) $20k - $40k

( ) $40k - $60k

( ) $60k - $80k

( ) $80k - $100k

( ) $100k or more

ID: 16

7) What is your involvement with the horse industry (check all that apply)?

[ ] Coach or riding instructor

[ ] Horse trainer

[ ] Farm, ranch, or stable owner/manager

[ ] Riding school student

[ ] Horse breeder

[ ] Pleasure rider

[ ] Competition athlete

[ ] Competition judge, official, or organizer

[ ] Volunteer for horse organization

[ ] Employee for horse organization

[ ] Horse product sales

[ ] Service provider

[ ] Horse racing

[ ] Horse rescue or sanctuary

[ ] Outfitting or trail riding

[ ] Veterinarian

[ ] Farrier

[ ] Animal health technician

[ ] Equine massage or chiropractic

[ ] Therapeutic or rehabilitation riding

[ ] Other, please list:: ____

[ ] Educator

ID: 17

8) How many years have you been involved with the horse industry?

( ) 0-5 years

( ) 6-10 years

( ) 11-20 years

( ) 21-30 years

( ) 30 years or more

Logic: Show/hide trigger exists.

ID: 18

9) Which disciplines are you involved with (check all that apply)?

[ ] Breed competitions

[ ] Halter classes

[ ] Natural horsemanship

[ ] Horse racing

[ ] Western pleasure

[ ] Cutting

[ ] Barrel racing

[ ] Cattle penning

[ ] Reining

[ ] Roping

[ ] Rodeo

[ ] Mounted shooting

[ ] English pleasure

[ ] Eventing

[ ] Dressage

[ ] Jumper

[ ] Hunter

[ ] Fox hunting

[ ] Endurance
[ ] Vaulting
[ ] Gymkhana
[ ] Saddleseat
[ ] Polo
[ ] Driving
[ ] Outfitting or trail riding
[ ] Other, please list:: ____

Logic: Hidden unless: Question "Which disciplines are you involved with (check all that apply)?" #9 is one of the following answers ("Breed competitions")

ID: 19

10) Please list the type(s) of breed competition(s) you participate in:

____

Logic: Show/hide trigger exists.

ID: 20

11) Are you affiliated with any horse organizations or clubs?

( ) Yes
( ) No

Logic: Hidden unless: Question "Are you affiliated with any horse organizations or clubs?" #11 is one of the following answers ("Yes")

ID: 21

12) Please list the name(s) of any horse organizations or clubs that you are affiliated with:

(eg. Equine Canada, Ontario Equestrian Federation, Horse Council of British Columbia, etc.)

____

ID: 23

13) How many hours per week do you spend directly with horses?

(eg. Grooming, riding, feeding, etc.)

( ) 0 hrs
( ) 5 hrs or less
( ) 6-15 hrs
( ) 16-35 hrs
( ) 35 hrs or more

ID: 24

14) How would you describe your current level of horse care knowledge?

( ) Not knowledgeable at all
( ) Somewhat knowledgeable
( ) Very knowledgeable
( ) Extremely knowledgeable

ID: 25

15) Are you aware of or knowledgeable about any of the following (check all that apply)?

[ ] Canadian Code of Practice for the Care and Handling of Equines
[ ] Body condition score (BCS)
[ ] American Association of Equine Practitioners (AAEP) Lameness Scale
[ ] The Five Freedoms
[ ] Equitation science
[ ] None of the above

Working with horses

Logic: Show/hide trigger exists.
ID: 40

19) Do you currently work with horses directly? ***(eg. Riding, driving, handling, etc.)***

( ) Yes
( ) No

Horse ownership

Logic: Show/hide trigger exists.
ID: 57

28) Do you currently own or have at least one horse in your care?

( ) Yes ( ) No

Logic: Hidden unless: Question "Do you <u>currently</u> own or have at least one horse in your care?" #28 is one of the following answers ("Yes")

ID: 58

29) How many horses do you currently own or have in your care?

( ) 1 horse
( ) 2-4 horses
( ) 5-10 horses
( ) 11-20 horses
( ) 20 horses or more

Management practices

For this section: please answer according to your current management practices if you are a horse owner or caregiver. If you do not currently own or care for a horse, please answer according to your perception of common industry practices in the Canadian horse industry.

Logic: Hidden unless: Question "Do you <u>currently</u> own or have at least one horse in your care?" #28 is one of the following answers ("Yes")

ID: 59

30) The <u>primary</u> purpose my horse(s) serves is:

( ) Pleasure or trail riding
( ) Racing competition
( ) Non-racing competition
( ) Riding or school lessons
( ) Work (eg. Ranching, logging, outfitting, etc.)
( ) Breeding
( ) Companionship (eg. Retired horse, etc.)
( ) Horse therapy or rehabilitation
( ) Other, please list:: ____

Human-horse interaction

ID: 108

79) Indicate how much you agree or disagree with the following statements:

	**Strongly agree**	**Agree**	**Neutral**	**Disagree**	**Strongly disagree**
Horses are livestock	( )	( )	( )	( )	( )
Horses are companion animals and/or pets	( )	( )	( )	( )	( )
Horses are friends, family, or children	( )	( )	( )	( )	( )
Horses can experience pain	( )	( )	( )	( )	( )
Horses can experience fear	( )	( )	( )	( )	( )
Horses can experience boredom	( )	( )	( )	( )	( )
Horses can experience frustration	( )	( )	( )	( )	( )
Horses can experience depression	( )	( )	( )	( )	( )
Horses can experience sadness	( )	( )	( )	( )	( )

Horses can experience jealousy	( )	( )	( )	( )	( )
Horses can experience anger	( )	( )	( )	( )	( )
Horses can experience happiness	( )	( )	( )	( )	( )
Horses can experience love	( )	( )	( )	( )	( )

Welfare issues

ID: 172

102) What is the best way to assess horse welfare?

Definition:
Time budget: tracking the amount of time an animal spends on various activities such as eating, sleeping, grooming, playing, etc.

( ) Vitals (eg. Heart rate, temperature, respiration rate, etc.)

( ) Coat condition

( ) Good performance

( ) Time budget

( ) Absence of abnormal behaviour

( ) Other, please list:: ____

Logic: Show/hide trigger exists.

ID: 268

104) Do you believe there are horse welfare issues in the Canadian horse industry?

**Definition:**
**Welfare:** overall physical and psychological well-being of an animal. Good welfare is a state wher animal is free from hunger or thirst; distress and discomfort; pain, injury, and disease; unnecessa suffering, and is able to express normal behaviour.

( ) Yes ( ) No

**Logic: Hidden unless: Question "Do you believe there are horse welfare issues in the Canadian horse industry?
#104 is one of the following answers ("Yes")**

ID: 175

105) Which horses are <u>at risk</u> for welfare issues (check all that apply)?

[ ] Horses at riding barns or stables

[ ] Horses at breeding facilities

[ ] Horses at rescue facilities or sanctuaries

[ ] Competition or performance horses

[ ] Horses on farms or acreages

[ ] Horses at auctions or feedlots

[ ] Other, please list:: ____

**Logic: Hidden unless: Question "Do you believe there are horse welfare issues in the Canadian horse industry?
#104 is one of the following answers ("Yes")**

ID: 179

106) Indicate how much you <u>agree or disagree</u> about whether the following are horse welfare issues:

	**Strongly agree**	**Agree**	**Neutral**	**Disagree**	**Strongly disagree**
Unwanted horses and what to do with them	( )	( )	( )	( )	( )
Horses that	( )	( )	( )	( )	( )

are not trained appropriately					
Inappropriate or lack of horse knowledge by owners or caregivers	( )	( )	( )	( )	( )
Financial costs of horse care	( )	( )	( )	( )	( )
Horse slaughter	( )	( )	( )	( )	( )
Not having the option of horse slaughter	( )	( )	( )	( )	( )
Ineffective or lack of horse welfare laws	( )	( )	( )	( )	( )
Ineffective or lack of enforcement capacity to protect horse welfare	( )	( )	( )	( )	( )

Logic: Hidden unless: Question "Do you believe there are horse welfare issues in the Canadian horse industry?
#104 is one of the following answers ("Yes")

ID: 188

107) Rank the following potential causes for reduced horse welfare, in terms of <u>frequency</u>:

(1=most frequent cause, 7=least frequent cause)

________Lack of financial resources by horse owners

________Pride or unwillingness to reach out for help

________Ignorance or lack of knowledge about welfare issues

________Traditional practice in horse industry or unwillingness to embrace new methods

________Malice or intention to hurt horses

________Greed or profit driven

________Lack of time by horse owner or staff

Logic: Hidden unless: Question "Do you believe there are horse welfare issues in the Canadian horse industry?
#104 is one of the following answers ("Yes")

ID: 190

108) Rank the following strategies for improving horse welfare, in terms of effectiveness:

(1=most effective strategy, 5=least effective strategy)

________Education programs about standards of horse care

________Stricter regulations and enforcement by horse organizations

________Stricter regulations and enforcement by governmental organizations

________Welfare certification programs for horse facilities

________Low stress and humane handling training for horse professionals

ID: 191

109) Who should deliver educational programs about horse care (check all that apply)?

[ ] Governmental organizations (eg. National Farm Animal Care Council, etc.)

[ ] Horse organizations (eg. Equine Canada, etc.)

[ ] Horse researchers

[ ] Veterinarians

[ ] Other, please list:: ____

Logic: Show/hide trigger exists.

ID: 192

110) Do you think a welfare scoring system, used to certify horse facilities as "welfare-friendly" is an effective strategy for improving or maintaining horse welfare?

( ) Yes

( ) No

**Logic: Hidden unless: Question "Do you think a welfare scoring system, used to certify horse facilities as "welfare-friendly" is an effective strategy for improving or maintaining horse welfare?" #110 is one of the following answers ("Yes")**
ID: 193

111) "Welfare-friendly" certification should be granted by:

( ) A governmental organization

( ) A horse organization

**Logic: Hidden unless: Question "Do you think a welfare scoring system, used to certify horse facilities as "welfare-friendly" is an effective strategy for improving or maintaining horse welfare?" #110 is one of the following answers ("Yes")**
ID: 194

112) What extra amount per month would you be willing to pay to board a horse(s) at a "welfare-friendly" certified facility?

( ) $0 (would not pay extra)

( ) $1-$50 per month

( ) $51-$100 per month

( ) $100 or more per month

Scenario questions

ID: 252

Please read the following scenarios. Move the slider along the line to indicate the level of welfare for a horse(s) in the following scenarios:

Validation: Min = 0 Max = 100

ID: 195

113) Horses are kept in a large pasture during the winter months. They are given continuous free access to a hay bale, and a new bale is provided before the hay runs out. The horses' primary water source is from eating snow.

0 _________________________[__]______________________________ 100

Validation: Min = 0 Max = 100

ID: 253

114) Scenario: A horse is housed individually in a box stall, and turned out individually into a small outdoor pen for a few hours each day. The horse can see other horses, but not interact with them. Good quality hay is provided three times a day in the stall, but no hay or grass is available in the outdoor pen.

0 _________________________[__]______________________________ 100

Validation: Min = 0 Max = 100

ID: 254

115) Scenario: A horse sustains a leg injury that requires long-term pain medication and two months of stall rest. The horse is given painkillers under veterinary supervision and is housed individually in a box stall, with one hand-led walk for 30 minutes per day.

0 _________________________[__]______________________________ 100

Validation: Min = 0 Max = 100

ID: 255

116) Scenario: At a barn, horses are stabled overnight and turned out into a large pasture for most of the day. The staff adds fresh straw to the stalls each day, but no manure or urine is picked out regularly, creating deep litter bedding.

0 _________________________[__]______________________________ 100

Validation: Min = 0 Max = 100
ID: 256

117) Scenario: A horse owner is experiencing financial difficulty. The owner compensates by prolonging trimming of the horses' hooves to every 10 weeks, deworms only in spring and fall, and feeds hay once a day.

0 _________________________[__]______________________________ 100

Validation: Min = 0 Max = 100
ID: 257

118) Scenario: A trainer uses a whip to discipline young horses for misbehavior. The horses are repetitively tapped lightly with a whip until they stop the unwanted behaviour. Once they stop, the horses are rewarded with a pat on the neck.

0 _________________________[__]______________________________ 100

Validation: Min = 0 Max = 100
ID: 258

119) Scenario: A horse is cribbing the top of their stall door. To stop the horse from cribbing, a horse owner puts a cribbing collar on the horse, and takes the collar off only when the horse is ridden.

0 _________________________[__]______________________________ 100

Validation: Min = 0 Max = 100
ID: 259

120) Scenario: A horse owner is backing (saddling) a young horse for the first time. To prevent any potential bucking or rearing, the owner administers a sedative to the horse prior to working with him.

0 _________________________[__]______________________________ 100

Comments

ID: 212

125) If you would like to express any concerns or insights about horse welfare issues in Canada, please feel free to provide comments below:

____

____

Thank You!
